# FAK Inhibition Induces Glioblastoma Cell Senescence-Like State through p62 and p27

**DOI:** 10.3390/cancers12051086

**Published:** 2020-04-27

**Authors:** Lía Alza, Mireia Nàger, Anna Visa, Carles Cantí, Judit Herreros

**Affiliations:** 1 Calcium Signaling Group, IRBLleida, University of Lleida, Rovira Roure 80, 25198 Lleida, Spain; lia.alza@udl.cat (L.A.); annavisa@mex.udl.cat (A.V.); carles.canti@udl.cat (C.C.); 2 Department of Medical Biology, UiT The Arctic University of Norway, 9010 Tromsø, Norway; mireia.nager@uit.no

**Keywords:** FAK, glioblastoma, proliferation, p62/SQSTM-1, p27/CDKN1B

## Abstract

Focal adhesion kinase (FAK) is a central component of focal adhesions that regulate cancer cell proliferation and migration. Here, we studied the effects of FAK inhibition in glioblastoma (GBM), a fast growing brain tumor that has a poor prognosis. Treating GBM cells with the FAK inhibitor PF-573228 induced a proliferative arrest and increased cell size. PF-573228 also reduced the growth of GBM neurospheres. These effects were associated with increased p27/CDKN1B levels and β-galactosidase activity, compatible with acquisition of senescence. Interestingly, FAK inhibition repressed the expression of the autophagy cargo receptor p62/SQSTM-1. Moreover, depleting p62 in GBM cells also induced a senescent-like phenotype through transcriptional upregulation of *p27*. Our results indicate that FAK inhibition arrests GBM cell proliferation, resulting in cell senescence, and pinpoint p62 as being key to this process. These findings highlight the possible therapeutic value of targeting FAK in GBM.

## 1. Introduction

Glioblastoma (GBM) is the most common malignant primary brain tumor. At present, it remains an incurable disease. This is due to its fast growth and infiltration into the brain parenchyma, which results in tumor recurrence. Consequently, novel therapies are needed to improve upon the current standard treatment, which consists of surgery, radiotherapy, and chemotherapy with temozolomide.

Focal adhesion kinase (FAK/PTK2) is a cytoplasmic tyrosine kinase that regulates the signaling cascades emanating from integrins and growth factor receptors. FAK modulates fundamental roles in cancer cells, such as cell proliferation, migration, and survival, and in the tumor microenvironment, for example, inducing angiogenesis [1,2]. The crucial involvement of FAK in cancer cell migration, invasion, and metastasis is well established [3]. By linking integrin signaling to the actin cytoskeleton, FAK controls the formation and disassembly of focal adhesions and cell migration dynamics [2,4,5,6].

Active FAK phosphorylated at Y397, a residue targeted and dephosphorylated by Phosphatase and Tensin homolog (PTEN) [7], is observed in GBM cell lines [8]. FAK activation promotes cell proliferation by accelerating G1 to S phase transition through increased expression of cyclin-D1/CCND1 and reducing p21/CDKN1A cyclin-dependent kinase (cdk) inhibitor levels [1,9,10]. Moreover, PTEN negatively regulates G1/S transition. This occurs by inhibiting the expression of S-phase kinase-associated protein-2 (SKP2), a component of the ubiquitin ligase Skp1, Cul1 and F-box (SCF) complex, and thus preventing the degradation of the cdk inhibitor p27/CDKN1B [11]. Thus, loss of *PTEN* in GBM supports FAK activation and resistance to apoptosis induced by the lack of cell–matrix contact.

Targeting of FAK has been considered in preclinical and clinical oncological trials [2,12]. Here, we used PF-573228, an inhibitor of the catalytic activity of FAK [2,13], to investigate its effects on GBM cell proliferation. FAK inhibition reduced GBM cell proliferation of adherent and GBM neurosphere cultures. Interestingly, PF-573228 increased p27/CDKN1B levels and β-galactosidase activity and decreased *p62/SQSTM-1* expression. We also found that p62-depleted cells transcriptionally upregulate *p27*. Therefore, p62 appeared negatively regulated in senescence-like cell cycle arrest.

## 2. Results

### 2.1. FAK Inhibition Reshapes GBM Cell Morphology, Increasing Cellular and Nuclear Size

**Figure 1:**
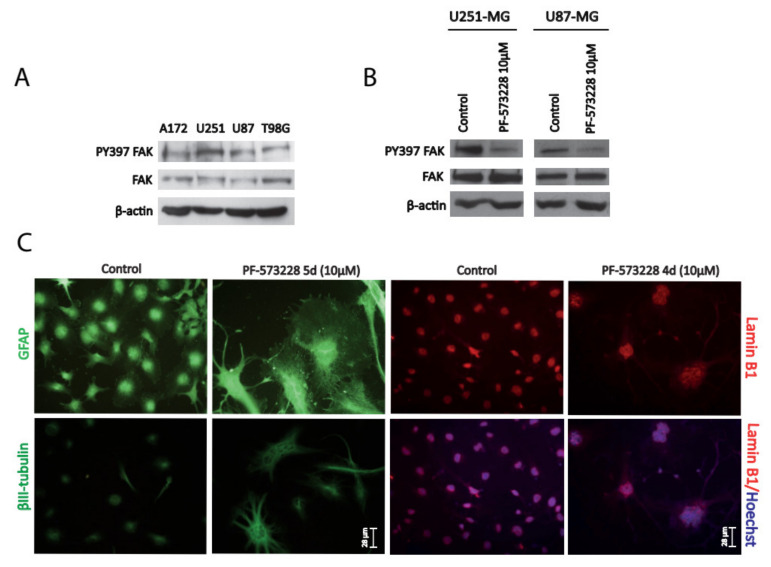
Inhibition of focal adhesion kinase (FAK) reshapes glioblastoma (GBM) cell morphology and increases cell size. (**A**) Cell lysates from different GBM cell lines (A172, U251-MG, U87-MG, and T98G) were analyzed for PY397 FAK and total FAK. β-actin was used as a loading control. GBM cell lines display active PY397 FAK, with U251-MG and U87-MG showing the highest levels. (**B**) U251-MG and U87-MG cell lysates (control or treated with PF-573228 10 µM) were analyzed for active and total FAK. β-actin was used as a loading control. FAK inhibitor effectively reduced PY397 FAK levels. (**C**) Glial Fibrillary Acidic Protein (GFAP), βIII-tubulin, and Lamin B1 immunostainings performed in U251-MG cells (after 4–5 days of treatment with PF-573228 10 µM). Cytoskeleton remodeling accompanied by cell body enlargement and lobulated/enlarged nuclei is revealed by Lamin B1 immunostaining. Bars = 28 µm.

We firstly analyzed the levels of total and active phosphorylated Y397 (PY397) FAK in cell lysates of different GBM cell lines. Active FAK was detected under basal conditions in the four GBM cell lines tested (Figure 1A). Cell lysates of three GBMs and two low-grade astrocytomas were also studied and FAK levels compared to those of mouse embryonic fibroblast (MEF) cell lysates. Total and active FAK levels were lower in GBMs compared with grade II gliomas or MEF (used as a control) (Figure S1A). This is consistent with in silico analysis of *FAK/PTK2* mRNA levels that confirmed a lower expression in GBM compared with astrocytoma biopsies (Figure S1B).

For the rest of the study, we used the GBM cell lines U251-MG and U87-MG, which displayed the highest levels of active FAK. Treatment of cells with PF-573228 (10 µM) for 24 hours resulted in the reduction of FAK activity, evidenced by decreased levels of PY397 FAK (Figure 1B), and severely altered their morphology (Figure S1D). Similar results were obtained with another FAK inhibitor, Defactinib (VS-6063/PF-04554878), at 5 µM (Figure S1 C and D). We confirmed a striking remodeling of the cytoskeleton (revealed by Glial Fibrillary Acidic Protein (GFAP) and βIII-tubulin immunostainings; Figure 1C) and increased cell size following treatment with PF-573228. Furthermore, Lamin B1 immunostaining highlighted larger lobulated nuclei following FAK inhibition (Figure 1C).

### 2.2. FAK Inhibition Reduces GBM Cell Proliferation

**Figure 2:**
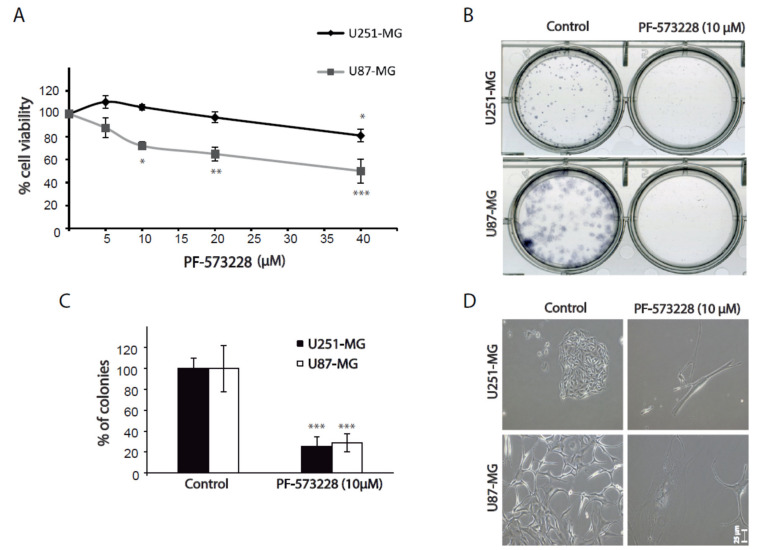
Inhibition of FAK reduces cell viability and clonogenic growth. (**A**) Cell viability assays performed in U251-MG and U87-MG cells treated with PF-573228 (from 5 µM to 40 µM) for 24 hours. Cell viability is significantly reduced from 10 µM in U87-MG cells and at 40 µM in U251-MG cells (one-way analysis of variance (ANOVA); **, *p* < 0.01, * *p* < 0.05; *** *p* < 0.001). (**B**) Clonogenic assays of GBM cell lines treated as indicated for 12–15 days. (**C**) Quantification of the number of cell colonies shows a decrease of 70% in the presence of FAK inhibitors compared with controls (***p < 0.001). (**D**) Representative phase contrast images of clonogenic assays showing control or PF-573228 treated cells. Bars = 25 µm.

Next, we studied whether FAK inhibition affected GBM cell proliferation. We firstly performed WST-1 viability assays in GBM cells treated with different concentrations of PF-573228 (from 5 to 40 µM) for 24 hours. The results showed a significant decrease in cell viability from 10 µM in U87-MG cells and at 40 µM in U251-MG (Figure 2A).

We also performed clonogenic assays to evaluate the capacity of cells to proliferate into clones. Cells grown in the presence of PF-573228 formed about 70% fewer cell colonies than untreated cells (Figure 2B,C). Again, cells treated with PF-573228 appeared strikingly flatter and larger than control cells (Figure 2D). WST-1 and clonogenic assays can reflect changes in both cell proliferation and survival. We did not observe significant cell death in GBM cells treated with FAK inhibitors. These results, therefore, suggest that FAK inhibition reduced cell proliferation.

**Figure 3:**
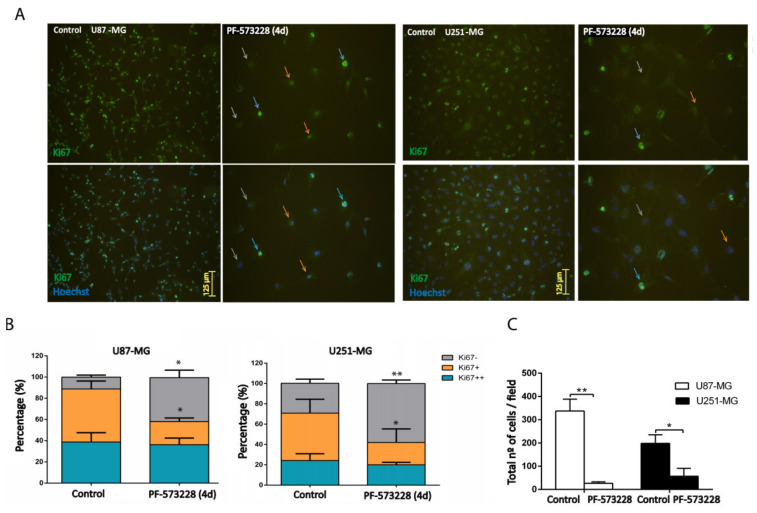
Inhibition of FAK reduces cell proliferation. (**A**) U87-MG and U251-MG cells (control or treated with PF-573228 10 µM for four days) were stained with Anti-Ki67 antibody. Nuclei were scored, according to the intensity of immunostaining, as Ki67++ (blue arrows), Ki67+ (orange arrows), and Ki67− (grey arrows). (**B**) Quantification of Ki67++, Ki67+, or Ki67− cells indicates that PF-573228 reduces the % of Ki67+ and increases Ki67− cells (28–25% decrease of Ki67+ and 30–29% increase of Ki67− in U87-MG and U251-MG cell lines, respectively; n = 3). Bars = 125 µm. (**C**) Plot shows the total number of Hoechst stained cells/field in each GBM cell line, which significantly decreases as a result of four days of treatment with 10 µM PF-573228 (** *p* < 0.01 and * *p* < 0.05). Bars = 125 µm.

To specifically address the question of FAK inhibition affecting cell proliferation, we performed immunostaining against Ki67, a marker expressed by proliferative cells. Ki67 protein levels vary along the cell cycle, being higher in the G2/M phase and lower in the G0/G1 phase [14]. We counted the number of cells showing high (Ki67++), medium (Ki67+), or low (Ki67−) immunoreactivity for Ki67 after four days of treatment with PF-573228. We found a decrease of ~25% in the number of Ki67+ cells and an increase of ~30% of Ki67− cells in both U87-MG and U251-MG cell lines (Figure 3A,B). At the same time, we observed a dramatic decrease in the mean number of cells/field after the four days of treatment (92% and 72% decrease compared with the control in U87-MG and U251-MG cell lines, respectively; Figure 3C).

**Figure 4:**
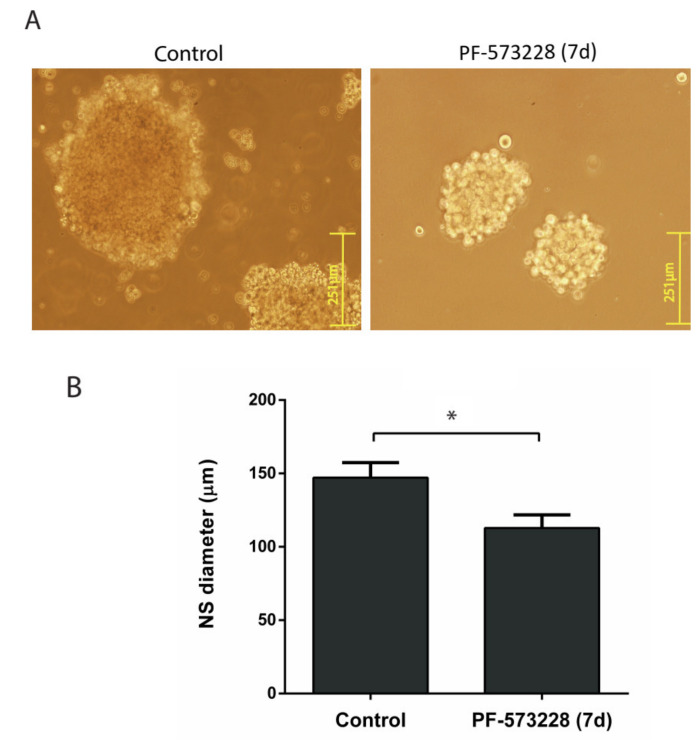
PF-573228 reduces neurosphere (NS) growth. (**A**) Representative images of U87-MG NSs grown for seven days in the presence or absence of PF-573228 (10 µM). (**B**) NSs grown in the presence of PF-573228 were smaller, with a reduction of 23% in their diameter (* *p* < 0.05; n = 4). Bars = 251 µm.

Finally, we investigated the effect of FAK inhibition on the growth of neurospheres (NSs), a model culture reflecting the proliferation of stem-cell-like cells. U87-MG derived NS grown for seven days in the presence of 10 µM PF-573228 showed a median diameter 23% shorter than that of the control NS (Figure 4).

We concluded that PF-573228 stops cell proliferation of different GBM cultures and increases Ki67− cells, possibly reflecting G0/G1 phase cells [14].

### 2.3. PF-573228 Increases p27 Protein Levels and β-Galactosidase Activity 

**Figure 5:**
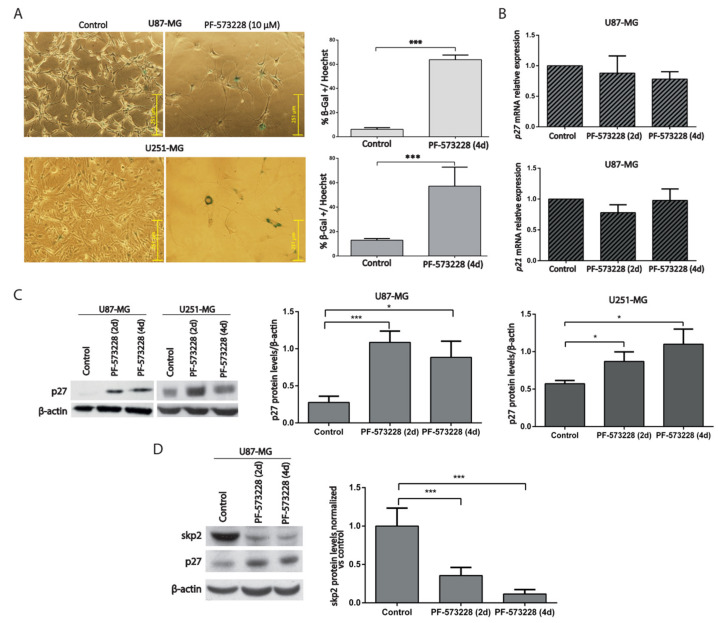
PF-573228 promotes GBM cell senescence. (**A**) Representative SA-β-gal staining from U87-MG and U251-MG cells, control or treated with PF-573228 10 µM for four days. The % of SA-β-gal positive cells significantly increases upon FAK inhibition (*** *p* < 0.001; n ≥ 3). Bar = 251 µm. (**B**) *p27/CDKN1B* and *p21/CDKN1A* mRNA levels did not change significantly between control cells or cells treated with PF-573228 (10 µM, two or four days; n = 3). (**C**) p27 protein levels were analyzed in control cells and cells treated PF-573228 for two or four days. β-actin was used as loading control. Quantification of p27 normalized to β-actin indicates that p27 significantly increases after two days of treatment with PF-573228, and after two and four days in U87-MG and U251-MG cells (* *p* < 0.05; n ≥ 4). (**D**) SKP2 protein levels were analyzed in control cells or cells treated PF-573228 for two or four days. β-actin was used as loading control. Plot represents SKP2 levels normalized vs. control, which decrease in PF-573228-treated cells (*** *p* < 0.001; n = 4).

Having observed the induction of cell cycle arrest and increased cell size, we decided to study the possible acquisition of a senescent phenotype following FAK inhibition. Firstly, we performed senescent associated β-galactosidase (SA-β-gal) staining on GBM cells, either control or treated with PF-573228, which reveals the increased activity of lysosomes at acidic pH typical of senescent cells [15]. Cultures treated with PF-573228 for four days showed a marked increase in the percentage of SA-β-gal-positive cells versus control cultures (58% and 44% increase in U87-MG cells and U251-MG, respectively; Figure 5A).

Analysis of Cdk inhibitors helps to identify senescent cells [16,17]. We, therefore, measured the mRNA levels of *p21/CDKN1A/CIP1* and *p27/CDKN1B/KIP1* in GBM cells after two or four days of treatment with PF-573228. The results showed no significant differences between control and treated cells (Figure 5B). However, p27 protein levels increased following treatment with PF-573228 in U87-MG and U251-MG cells (Figure 5C). These results suggest that PF-573228 stops GBM cell proliferation by stabilizing p27 protein.

SKP2 is a ubiquitin ligase that targets p27 [18] and is regulated by FAK [19,20]. We investigated whether SKP2 was modulated by PF-573228. We confirmed reduced SKP2 levels in parallel with increased p27 levels after PF-573228 treatment (Figure 5D). Therefore, treatment with PF-573228 increases both SA-β-gal staining and p27 protein in GBM cells.

### 2.4. p62/SQSTM-1 Expression is Reduced upon FAK Inhibition

**Figure 6:**
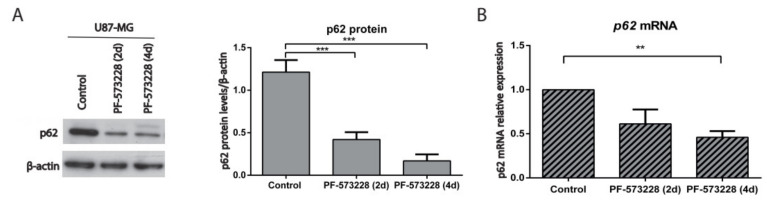
*p62* is repressed upon FAK inhibition. (**A**) p62 was analyzed by Western blot in control cells or cells treated with FAK inhibitors and β-actin was used as a loading control. Plot shows p62 protein levels normalized to β-actin and indicates that p62 significantly decreases after treatment with PF-573228 (10 µM, two and four days; n = 10) compared with untreated cells. (**B**) *p62* mRNA relative expression in control U87-MG cells and cells treated with PF-573228 (10 µM, two or four days). *p62* mRNA levels decrease after FAK inhibition (** *p* < 0.01; *** *p* < 0.001; n = 4).

P62/SQSTM-1 links autophagy and activation of different signaling pathways during tumorigenesis [21]. Its best-known role in autophagy is that of a cargo receptor of autophagosomes, although it can also be a substrate. Moreover, p62 is phosphorylated by Cdk1 to achieve optimal transition through mitosis [22]. PF-573228 significantly reduced p62 protein levels compared with untreated cells (Figure 6A). This result could be explained by its degradation through autophagy or by transcriptional repression. To clarify these possibilities, the mRNA levels of *p62* were measured by real-time qPCR. Interestingly, we observed that *p62* expression decreases upon PF-573228 treatment (Figure 6B). We confirmed this finding using Defactinib, which increased the percentage of SA-β-gal-positive GBM cells in parallel to decreasing *p62* expression (Figure S2), similar to PF-573228. These findings suggest that the decay of p62 associates with the proliferative arrest promoted by FAK inhibition, consistent with a pro-neoplastic role of p62 [22,23,24].

### 2.5. Depletion of p62 Increases p27 and SA-β-Gal Activity

**Figure 7:**
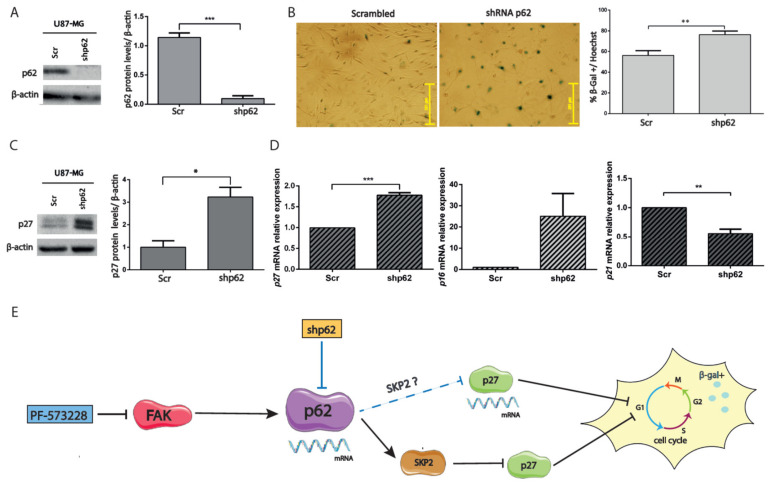
Silencing *p62* increases *p27* expression. (**A**) Cell lysates of U87-MG cells expressing scrambled (Scr) or p62 shRNAs (shp62) were immunoblotted for p62 and compared with β-actin, used as loading control. Plot represents the p62 levels in cells expressing shRNAs of p62 or Scr (*** *p* < 0.001; n = 4). (**B**) Representative SA-β-gal staining performed in cells expressing Scr or p62 shRNAs. Plot represents the % of SA-β-gal compared with the total number of cells stained by Hoechst (** *p* < 0.01; n = 3). Bars = 251 µm. (**C**) Cell lysates of cells expressing Scr or p62 shRNAs were immunoblotted for p27 and β-actin. p27 levels (normalized to β-actin) increase in p62-depleted cells (* *p* < 0.05; n = 4). (**D**) *p27/CDKN1B*, *p21/CDKN1A*, and *p16/CDKN2A* mRNA levels were measured from cells expressing Scr or p62 shRNAs. *p27* expression significantly increases, whereas *p21* decreases, in p62-depleted cells (*p16* expression was upregulated in two of three experiments). *** *p* < 0.001 and ** *p* < 0.05 (n ≥ 3). (**E**) Proposed model: PF-573228 transcriptionally downregulates *p62,* decreases SKP2, and post-transcriptionally elevates p27. Both FAK inhibition and *p62* silencing (blue lines) increase SA-β-gal activity (represented with an enlarged cell, arrested cell cycle, and β-gal positivity), in the latter case through transcriptional upregulation of *p27.*

To further understand the involvement of p62 in the proliferation arrest obtained upon inhibition of FAK, we used U87-MG cells in which *p62* expression had been silenced (by shRNA technology) and analyzed senescence markers, SA-β-gal, and Cdk inhibitors. GBM cells depleted of p62 showed altered morphology and increased SA-β-gal staining compared with control cells (expressing scrambled shRNA; Figure 7A and Figure 7B). These findings were accompanied by increased p27 protein and mRNA levels and by decreased *p21* mRNA levels (Figure 7C and Figure 7D). In conclusion, FAK inhibition stops GBM cell proliferation through p27 and results in a senescent-like phenotype associated with reduced *p62* expression. Conversely, silencing *p62* transcriptionally upregulates *p27*.

## 3. Discussion

We investigated the effects of FAK inhibition in GBM cell proliferation. Our results in cell viability, clonogenic, and Ki67 immunostaining experiments indicate that the cell cycle is arrested upon treatment with PF-573228. The proliferative arrest occurs through increased p27 protein levels (p27 or p21 mRNA levels remain unaltered) and phenotypically correlates with a flattened cell body and SA-β-gal positivity, suggesting senescence entry. Interestingly, *p62* is repressed by FAK inhibitors, PF-573228 and Defactinib. This finding prompted us to analyze p62-depleted cells, which transcriptionally upregulate *p27* and increase SA-β-gal activity. Our results, therefore, indicate that *p62* downregulation is associated with senescent phenotypes. In fact, p62 has recently been associated with longevity and its absence to aging in *C. elegans* [25]. We propose that FAK inhibition may be a valid strategy to counteract GBM progression through senescence deregulation. 

Senescence is a cell cycle arrest program controlled by different Cdk inhibitors depending on the senescence trigger. It has been linked to Lamin B1 loss [26] (occurring through autophagy in oncogenic senescence [27]) and to a secretory phenotype [16,28]. It is related to an enlarged flat morphology and hypertrophy [29] supported by extensive cytoskeletal changes [30]. Importantly, senescent cells appear to contain smaller focal adhesion contacts with hypophosphorylated FAK, which could account for their impaired proliferation and migration capacities [30]. The GBM cell proliferative arrest observed upon pharmacological inhibition of FAK appears critically regulated by p27, a Cdk inhibitor involved in therapy-induced senescence (TIS) [16,31]. However, p27 has also been associated with cell quiescence [28]. Analysis of additional senescent markers like those involved in a secretory phenotype or in TIS awaits future research and should help clarifying the observed cell phenotype. Senescence-like induction through the p27 pathway may be a consequence of defects in the p53 and p16 pro-senescence pathways in GBM and could be exploited to overcome the *PTEN* loss in this tumor [11,32].

FAK inhibitors reduce cell proliferation, induce apoptosis, and slow GBM growth in vitro [8] and in vivo [33]. Specifically, PF-573228 promotes proliferative arrest through decreased CyclinB1 and Lamin A/C, and induces cancer cell senescence [34]. The effects of PF-573228 in GBM cell proliferation were linked to increasing numbers of Ki67-negative cells and to the stabilization of p27, as a result of SKP2 downregulation [11,19,32]. These results are consistent with the finding that the inactive Y397F FAK mutant reduces proliferation by reducing the levels of cyclins (D1 and E) and increasing those of p27 and p21 [9]. In contrast, the effects of PF-573228 seem independent of the p53-p21 axis [16] in GBM. We did not observe apparent cell death upon PF-573228 treatment. Yet, effects on apoptosis in U87-MG cells cannot be ruled out as they were described for other FAK inhibitors [8,33]. Finally, the pluripotency gene Nanog that regulates the proliferation of glioma stem cells [35,36] is activated through phosphorylation by FAK [37], and its inhibition could explain the effects of PF-573228 on neurosphere growth.

Autophagy is a catabolic process allowing the degradation of proteins and damaged organelles. The relationship between autophagy and senescence is complex. While autophagy induction supports cell quiescence [38], impaired autophagy is considered a senescence driver [28,29,38]. Thus, decreased selective autophagy inhibits the degradation of proteins required in senescence such as GATA4 [39]. Previously, FAK depletion was linked to autophagy through the targeting of active Src to autophagosomes [40]. Here, we studied the adaptor protein p62, a central autophagy player and signaling modulator [21], upon FAK inhibition. P62 levels decrease following FAK inhibition, both by PF-573228 and Defactinib, resulting in a cell cycle arrest compatible with cell senescence. P62 is overexpressed in cancer, including GBM [41]. Indeed, several studies presented tumorigenic roles for p62 [23,24,42,43], while *p62* knockdown reduced Ki67 immunostaining and esophageal carcinoma growth [44]. In addition, p62 phosphorylated by Cdk1 is involved in the control of mitosis [22]. P62 upregulates SKP2, at both the mRNA and protein levels, through PKCiota and the proteasome system [44,45]. Furthermore, the p62/SKP2 axis promotes p21 and p27 degradation [45]. Importantly, we found that *p62* gene silencing upregulates *p27* expression, triggering a senescent phenotype. Salazar et al. also observed senescence of vascular smooth cells upon silencing *p62* [46]. Decreased p62 would lead to reduced amounts of SKP2, resulting in the stabilization of p27 in addition to the regulation of *p27* transcripts demonstrated here. P62 nuclear functions are ill-defined in spite of its nuclear localization signals [21] and nuclear shuttling [47]. Thus, the mechanisms by which p62 can modulate p27 expression remain unidentified. Our findings concerning the pharmacological inhibition of FAK with PF-573228 or the silencing of *p62* highlight the importance of p62 in cell senescence through p27 (Figure 7E). How FAK inhibitors can regulate p62 expression remains unclear. Identified binding sides on the *p62* promoter including those for AP-1 or NRF2 [21] could be potentially involved. Further studies are needed to clarify the integration of p62 in FAK signaling. Collectively, we observed a proliferative arrest indicative of senescence linked to *p62* repression after FAK inhibition. Our findings could be exploited by targeting FAK alone or in combination with temozolomide [33]. In addition, FAK inhibitors could be combined with senolytic agents in order to eliminate senescent-like cancer cells, which have been linked to inflammation and recurrence, to reduce GBM progression. Nevertheless, the implementation of preclinical models is the necessary next step to validate FAK as a valuable chemotherapeutic target in GBM. While in vitro data show that FAK inhibitors have an interesting profile against GBM, an important caveat is their bioavailability in the brain. Blood–brain barrier (BBB) permeability and brain efflux index are unknown for these compounds, so whether they can achieve clinically relevant concentrations in GBM tumors is a conundrum. Although the BBB is disrupted in GBMs, in some tumor regions, it can be intact and effectively preclude drug delivery [48]. The BBB permeability to chemotherapeutics in GBM is an active area of research, and different strategies are being investigated in order to enhance drug delivery [49]. Thus, the translationality of the findings reported here needs to be tested in vivo in GBM models, using patient-derived GBM cells and carefully monitoring the effective penetration of the FAK inhibitor into the brain.

## 4. Material and Methods

### 4.1. Reagents and Antibodies

Reagents were from the following companies: Hoechst (B2261), MTT (M2128), PF-573228 (PZ0117), polybrene (H9268) puromycin (P7225), and X-Gal (B4252) from Merck Sigma-Aldrich (Darmstadt, Germany); and Defactinib (VS-6063, PF-04554878) from Selleckchem (Houston, TX, USA).

Antibodies against the following proteins were used: PY397 FAK/PTK2 (Cell Signaling Danvers, MA, USA; 8556P), FAK (Cell Signaling 13009P), β-actin/ACTB (Merck Sigma-Aldrich A5441), βIII-tubulin/TUBB3 (Covance, Princetown, New Jersey, USA; PRB-435P), GFAP (Millipore, Burlington, MA, USA; AB5804), Ki67/MKI67 (Santa Cruz Biotechnology sc-15402), Lamin B1/LMNB1 (Calbiochem, San Diego, CA, USA; NA12), p27 (Genetex, Alton Pkwy Irvine, CA, USA; GTX100446-25), p62 (Novus Biologicals, Centennial, CO, USA; NBP1-48320), and SKP-2 (Abcam, Cambridge, UK; ab19877).

### 4.2. Cell Culture

GBM cell lines were obtained from American Tissue Culture Collection (ATCC) and maintained in minimal essential medium (Thermo Fisher Scientific, Waltham, MA, USA; 21090022) containing 10% heat-inactivated fetal bovine serum (FBS; Thermo Fisher Scientific 10270098), penicillin/streptomycin (Thermo Fisher Scientific 15140-122), L-glutamine (Thermo Fisher Scientific, 25030-081), 1% non-essential aminoacids (Thermo Fisher Scientific 11140-035). U87-MG (ATCC), and U251-MG cell lines were authenticated by short tandem repeat profiling (Stab Vida, Portugal) following purification of genomic DNA using the Maxwell16 Tissue DNA kit (Promega, Madison, Wisconsin; AS1030). Cell lines were grown in mycoplasma-free rooms and mycoplasma testing was performed by PCR (primers used were forward: GGCGAATGGGTGAGTAACACG and reverse: CGGATAACGCTTGCGACCTATG). Cells that tested positive were either discarded or treated with Plasmocin (Thermo Fisher Scientific ant-mpt-1). Cell lines were passaged for 20–25 passages. Primary GBM cell cultures were isolated as previously described [41] from surgical biopsies obtained from Hospital Arnau de Vilanova of Lleida (Spain), following approval by the review board of the IRBLleida Biobank and of the ethical committee of the University of Lleida (code 235/CEIC/2019).

Neurospheres (NSs) were grown in suspension from adherent U87-MG cells using Neurobasal media (Thermo Fisher Scientific LS21103049) containing B27 supplement (1:50; Thermo Fisher Scientific 17504044), penicillin/streptomycin, L-glutamine, 20 ng/mL basic Fibroblast Growth Factor (Thermo Fisher Scientific 13256029), and 20 ng/mL Epidermal Growth Factor (Thermo Fisher Scientific PHG0314), with or without PF-573228 for 5–7 days. Media was refreshed after three days of plating. The long diameter of NS was measured using Image J (20–45 spheres were measured in each experiment, n = 3).

### 4.3. Immunoblot Analysis

Cells were washed with PBS and lysed in Tris 62.5 mM, pH 6.8, and 2% sodium dodecyl sulfate (SDS). Cell lysates were separated by SDS-polyacrylamide gel electrophoresis and gels were transferred to a polyvinylidene difluoride (PVDF) membrane (Merck Millipore IPVH00010). Membranes were cut to probe different antibodies on the same membrane. Membranes were blocked with 5% milk and incubated overnight with primary antibodies. Blots were developed using enhanced chemiluminiscence (ECL Western Blotting Substrate, Fisher Thermo Scientific 32106) or Immobilon Forte Western Horse Radish Peroxidase substrate (Merck-Millipore WBLUF0100). Band intensity was measured using ImageJ software and normalized against β-actin. 

### 4.4. shRNA-Induced Gene Silencing by Lentiviral Infection

Lentiviral-based vectors pLKO.1-puro were used for RNA interference-mediated gene silencing, containing short hairpin RNAs (shRNAs) scrambled (against the sequence 50 CAACAAGATGAAGAGCACCAA 30) or against human *p62/SQSTM1* (Mission RNA, Merck Sigma-Aldrich, TRCN0000007237). Lentiviral particles were produced in HEK293T (human embryonic kidney) cells for 72 h upon transfection with shRNA vectors, together with psPAX2 and pMD2G plasmids using polyethylenimine. Medium was then centrifuged at 2,500 rpm and filtered through a 0.45 mm membrane. Cells were incubated with medium containing lentiviral particles together with polybrene (1.75 mg/mL) for 24 h. Medium was replaced after 24 h and cells cultured for seven days to allow the knockdown. Puromycin (2 µg/mL) was added to media to select for resistant cells and refreshed after three days.

### 4.5. Real-Time PCR

RNA was isolated using EZNA Total RNA Kit I (VWR, R6834-01). RNA (1 µg) was reverse transcribed (25 °C for 10 min, 42 °C for 1 h, and 92 °C for 5 min) using RevertAid RT Reverse Transcription Kit (VWR, Radnor, PA, USA; K1622). cDNA was then analyzed by quantitative PCR (qPCR) on a CFX96^TM^ Real-Time PCR Detection System (Bio-Rad, Hercules, CA, USA) using TaqMan hydrolysis probes labeled with FAM and TaqMan^TM^ Gene Expression Master Mix (Thermo Fisher Scientific, 10525395). Gene-specific probes used were human *p62/SQSTM1* (Hs00177654_m1), *p21/CDKN1A* (Hs01121172_m1), *p16/CDKN2A* (Hs00923894_m1), *p27/CDKN1B* (Hs00153277_m1), and *GAPDH* (used as internal control, Hs99999905_m1) from Thermo Fisher Scientific. Samples were assayed in triplicate for each gene and the relative expression was calculated by the ΔΔCt method (Applied Biosystems) and plotted.

### 4.6. Cell Viability Assay 

Cell viability was measured by the WST-1 (4-[3-(4-iodophenyl)-2-(4-nitrophenyl)-2H-5- tetrazolium]-1,3-benzene disulfonate; Roche, 05015944001) colorimetric assay. Cells were plated on 96-well plates (5,000 cells/well), treated for 24 h, and incubated for 60–120 min with 0.5 mg/mL WST-1 after 48 h of plating. Absorbance was measured at 440 nm, using a reference filter at 620 nm, in a microplate reader (Bio-Rad). Viability assays were performed in at least three independent experiments using triplicate measurements per condition.

### 4.7. Clonogenic Assays

For clonogenic assays, 500 cells/well were plated in six-well plates, grown for 12–15 days in the absence or presence of inhibitor, and stained with MTT (3-(4,5-Dimethylthiazol-2-yl)-2,5-Diphenyltetrazolium Bromide, 0.5 mg/mL). Cell colony numbers were counted using ImageJ software.

### 4.8. Immunohistochemistry

Cells were plated on Poly-D-Lysine (PDL)-coated (25 µg/mL) coverslips and treated with PF-573228 (10 µM). Treatments were performed for 2 or 4–5 days (refreshing treatments at day 2), as indicated. Cells were fixed using 4% Paraformaldehyde (20 min, room temperature (RT)); washed with phosphate buffered saline (PBS); permeabilized with Triton X-100 0.2% for 4 min; and blocked in 5% FBS, 5% horse serum, and 0.2% glycine in PBS. Cells were incubated with primary antibodies (overnight, 4 °C) and subsequently washed and incubated with Alexa Fluor 488 or 594 secondary antibodies (Thermo Fisher Scientific) and Hoechst. Coverslips were mounted on Mowiol and images were obtained using an inverted Olympus IX70 microscope (10×, 0.3 numerical aperture (NA); 20×, 0.4 NA; 32×, 0.4 NA) equipped with epifluorescence optics and a camera (Olympus OM-4 Ti). DPM Manager Software was used to manage the pictures. Ki67 immunoreactivity was quantified using ImageJ by counting the cells according to the intensity of the Ki67 immunostaining and compared with the total number of nuclei stained by Hoechst.

### 4.9. Senescence-Associated β-Galactosidase 

Cells were washed in PBS (pH 7.4), fixed for 4 min with 0.5% glutaraldehyde in PBS, and washed with 2 mM MgCl_2_ in PBS solution. Cells were then incubated with fresh senescence-associated stain solution (20 mg/mL X-Gal, 5 mM K_3_Fe(CN)_6_, 5 mM K4Fe(CN)_6_, and 2 mM MgCl_2_ in PBS (pH 6.0)) for 6–8 h at 37 °C. Cell nuclei were counterstained with Hoechst, pictured, and counted. Plots represent the % of SA-β-gal positive cells compared with the total number of cells stained by Hoechst (>100 control cells/field and 20–60 treated cells/field from five fields were counted from at least three independent experiments).

### 4.10. Statistical Analyses and Bioinformatics

Statistical significance was assessed by performing one-way analysis of variance (ANOVA) test (as indicated) or Student’s *t*-test. Asterisks represent different significance levels (* *p* < 0.05; ** *p* < 0.01; and *** *p* < 0.001). Experiments are represented as mean ± SEM (n ≥ 3). Expression analysis of FAK mRNA levels in non-tumoral, astrocytoma, and GBM samples was performed using Gliovis [50].

## 5. Conclusions

FAK inhibitor PF-573228 stops GBM proliferation, leading to p27 stabilization. Furthermore, FAK inhibition represses *p62/SQSTM-1* and increases senescence-associated β-galactosidase activity. Thus, FAK inhibition could be considered in novel therapies against GBM.

## Supplementary Materials

The following are available online at https://www.mdpi.com/2072-6694/12/5/1086/s1, Figure S1. (A) Total FAK and PY397 FAK levels were analyzed in GBM cultures (grade IV), low grade astrocytomas (grade II) compared with mouse embryonic fibroblasts (MEFs). FAK levels decrease in GBMs compared with astrocytomas or MEFs. (B) In silico analysis using Gliovis (Rembrandt dataset) confirms that GBMs express less *FAK/PTK2* mRNA than astrocytomas (** *p* = 0.01, Tukey’s test). (C) U251-MG and U87-MG cell lysates (control or treated with Defactinib 5 µM) were analyzed for active and total FAK. β-actin was used as a loading control. Defactinib effectively reduced PY397 FAK levels. (D) Representative phase contrast images of U87-MG and U251-MG cells control and treated with FAK inhibitors for 4–5 days. Bars = 100 µm. Figure S2. (A) Representative SA-β-gal staining from control U87-MG cells or treated with Defactinib (5 µM, 5 days). Bars = 251 µm. Plot represents the % of SA-β-gal positive cells, which significantly increases upon FAK inhibition (** *p* < 0.01; n = 3). (B) p62 was analyzed by Western blot in control cells or cells treated with Defactinib (5 µM, 5 days) and β-actin was used as a loading control. Plot shows p62 protein levels normalized to β-actin, indicating decreased p62 levels in both cell lines after Defactinib treatment (*** *p* < 0.001; n ≥ 3). (C) *p62* mRNA relative expression in control U87-MG cells and cells treated with Defactinib. *p62* mRNA levels decrease after FAK inhibition (** *p* < 0.05; n = 3).
