# Supplementary material for: FAK Inhibition Induces Glioblastoma Cell Senescence-Like State through p62 and p27
Source: Cancers (Basel). 2020 Apr 27;12(5):1086. doi: 10.3390/cancers12051086 (PMC7281094; doi:10.3390/cancers12051086)

Supplemental Materials

FAK Inhibition Induces Glioblastoma Cell Senescence-Like State through p62 and p27

Lía Alza, Mireia Nàger, Anna Visa, Carles Cantí and Judit Herreros

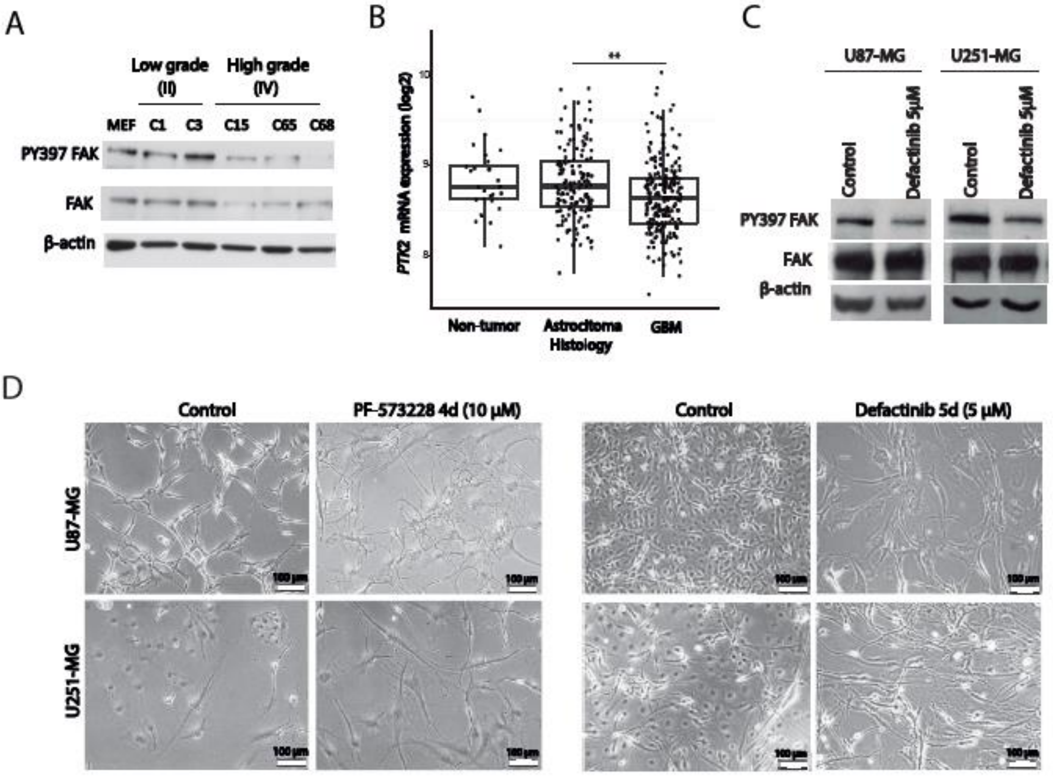

Figure S1. (**A**) Total FAK and PY397 FAK levels were analyzed in GBM cultures (grade IV), low grade astrocytomas (grade II) compared to mouse embryonic fibroblasts (MEF). FAK levels decrease in GBMs compared to astrocytomas or MEFs. (**B**) *In silico* analysis using Gliovis (Rembrandt dataset) confirms that GBMs express less *FAK*/*PTK2* mRNA than astrocytomas (** $p = 0.01$; Tukey's test). (**C**) U251-MG and U87-MG cell lysates (control or treated with Defactinib 5 μM) were analyzed for active and total FAK. β-actin was used as a loading control. Defactinib effectively reduced PY397 FAK levels. (**D**) Representative phase contrast images of U87-MG and U251-MG cells control and treated with FAK inhibitors for 4–5 days. Bars = 100 μm.

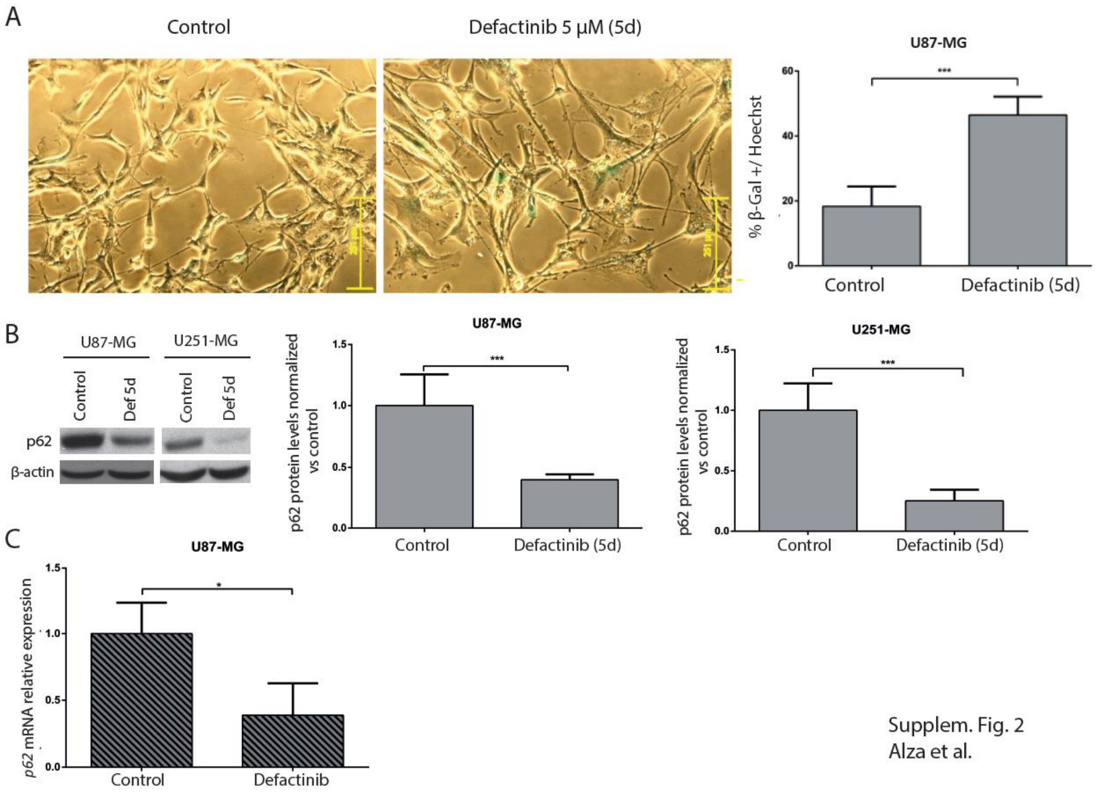

Figure S2. (**A**) Representative SA-β-gal staining from control U87-MG cells or treated with Defactinib (5 μM, 5 days). Bars = 251 μm. Plot represents the % of SA-β-gal positive cells, which significantly increases upon FAK inhibition (** $p < 0.01$; n = 3). (**B**) p62 was analyzed by Western-blot in control cells or cells treated with Defactinib (5 μM, 5 days) and β-actin was used as a loading control. Plot shows p62 protein levels normalized to β-actin, indicating decreased p62 levels in both cell lines after Defactinib treatment (*** $p < 0.001$; n ≥ 3). (**C**) *p62* mRNA relative expression in control U87-MG cells and cells treated with Defactinib. *p62* mRNA levels decrease after FAK inhibition (** $p < 0.05$; n = 3).

Uncropped western blots

Figure 1

A

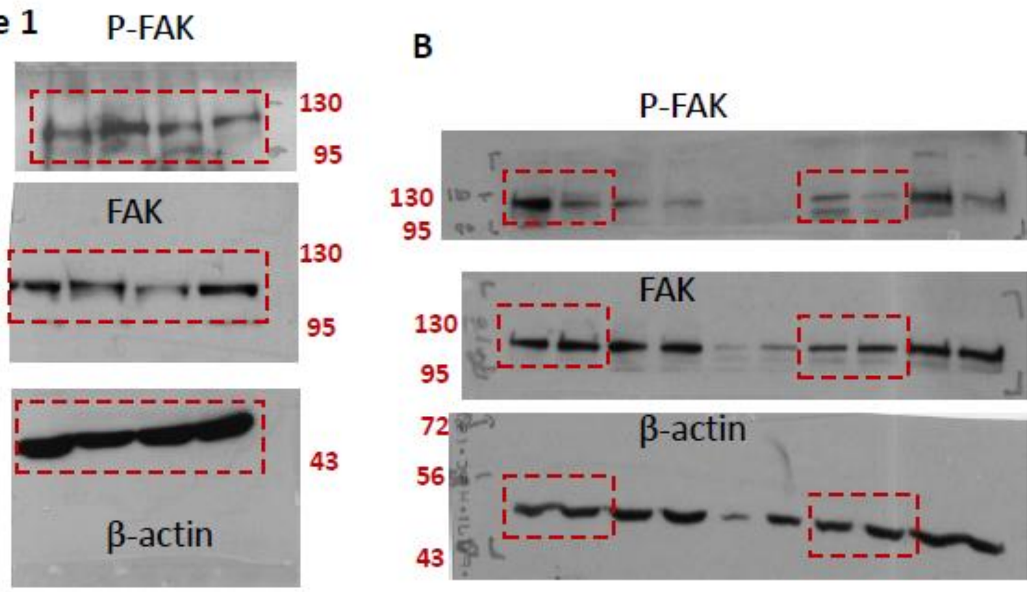

Figure 5

C

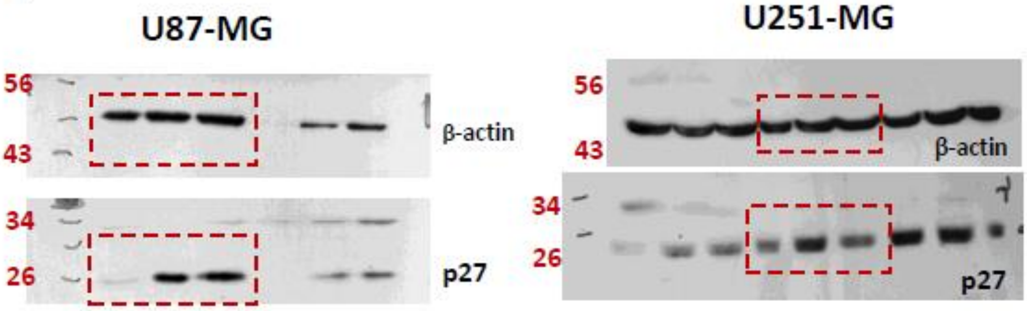

D

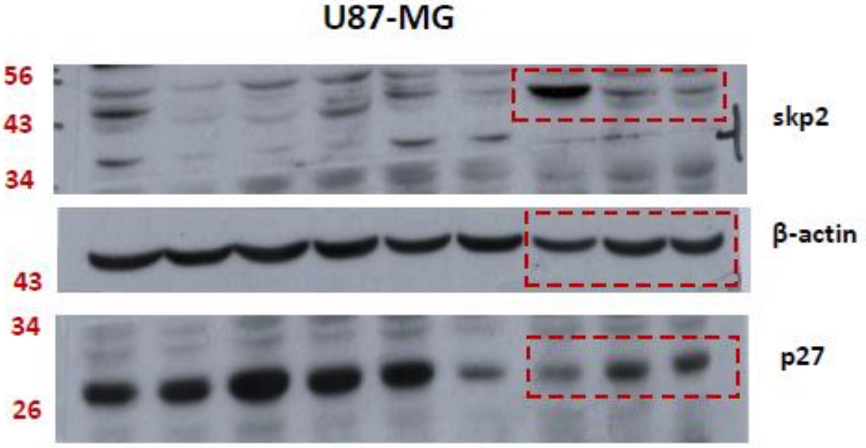

Figure 6

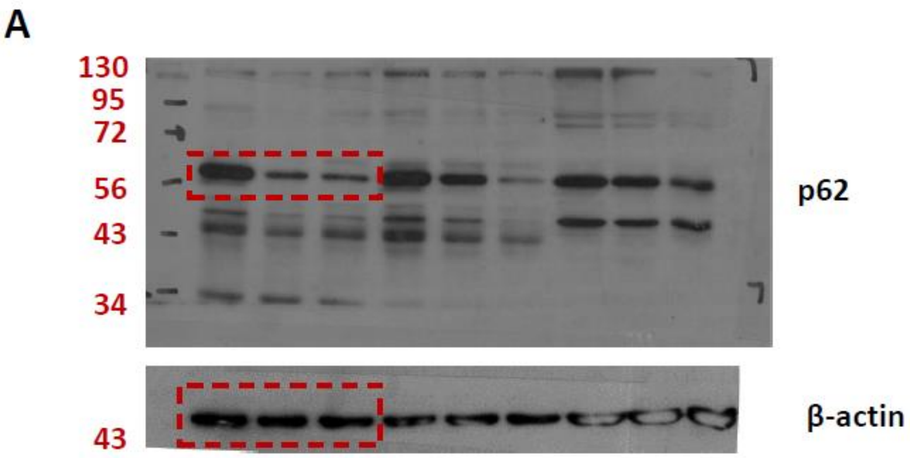

Figure 7

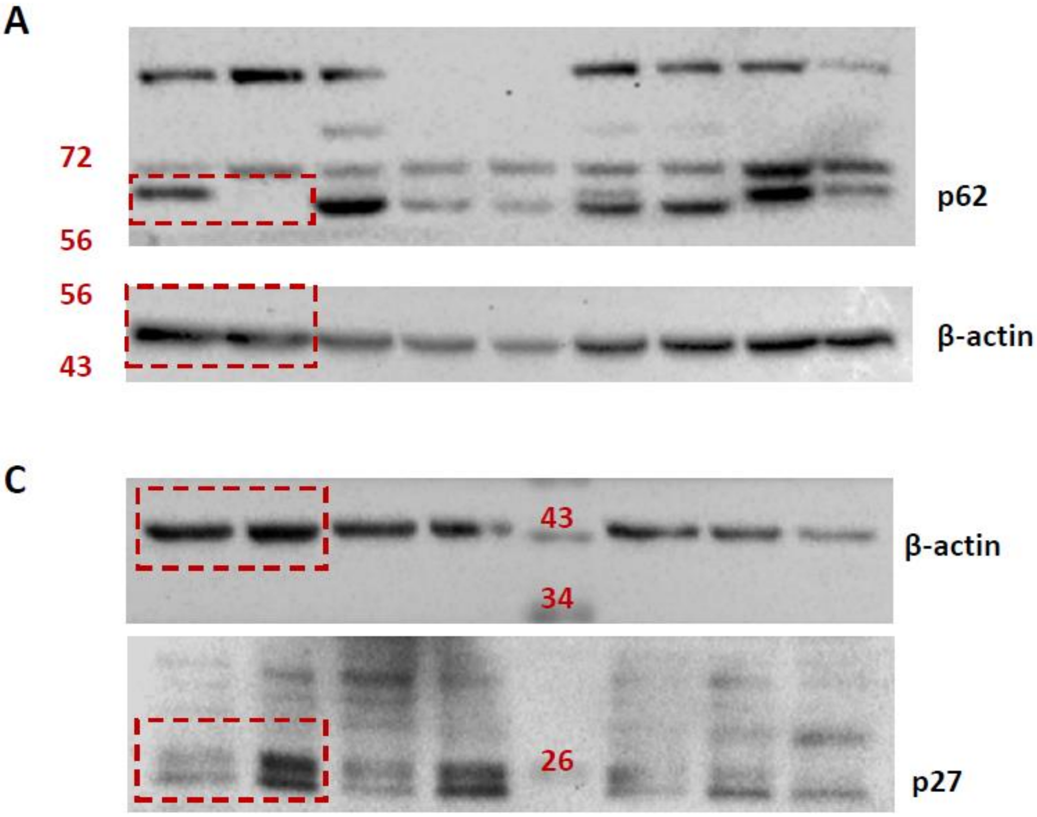

Figure Suppl. 1

A

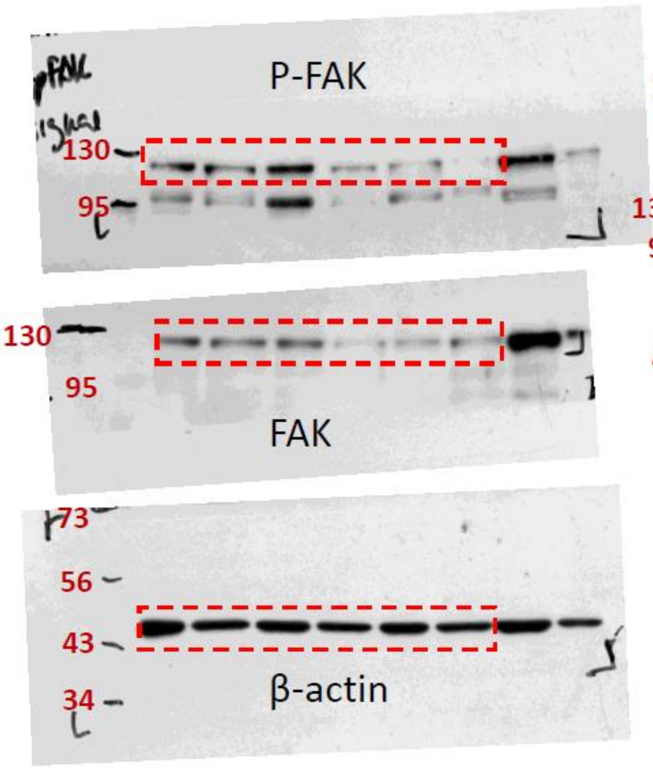

B

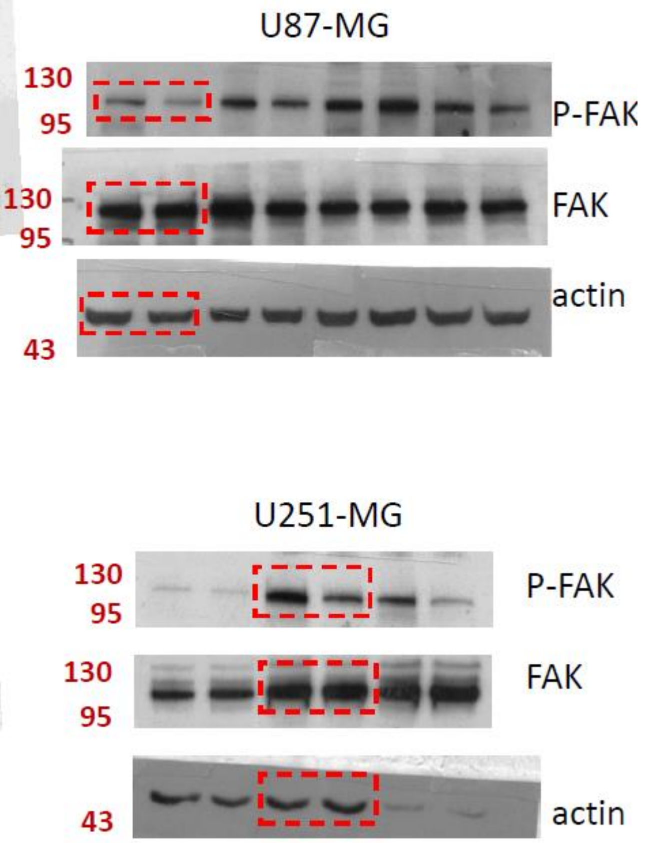

Figure Suppl. 2

B

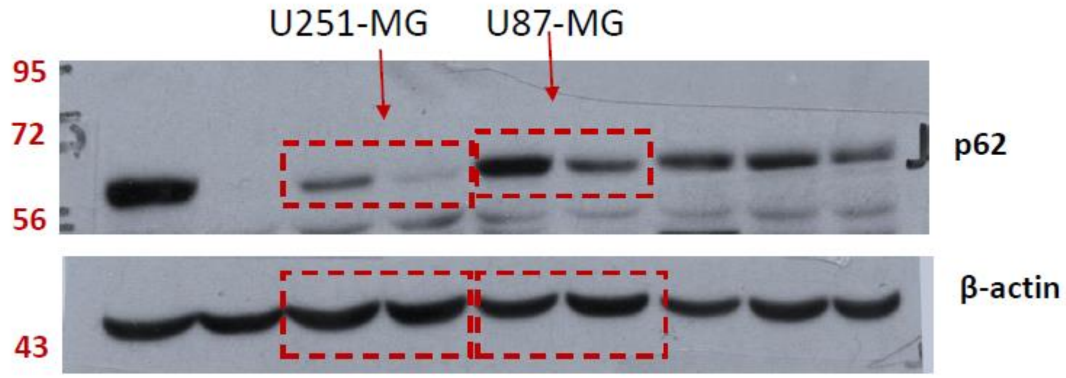

Supplement: Supplementary file 1 [file cancers-12-01086-s001.pdf]
